# A Case of Segmental Absence of Intestinal Musculature Associated With a Gastric Diverticulum in an Adult

**DOI:** 10.7759/cureus.100787

**Published:** 2026-01-04

**Authors:** Yuki Kubo, Naoko Nambu, Tsukasa Yoshikawa, Kaito Muroki, Masumi Kawashima, Koichi Hyodo, Nobuyuki Terada, Shin-ichi Nakatsuka

**Affiliations:** 1 Department of Pathology, Yao Tokushukai General Hospital, Yao, JPN; 2 Department of Surgery, Yao Tokushukai General Hospital, Yao, JPN; 3 Department of Radiology, Yao Tokushukai General Hospital, Yao, JPN

**Keywords:** adult, diverticulum, gastrointestinal congenital anomaly, segmental absence of intestinal musculature, stomach

## Abstract

A man in his late 50s underwent an abdominal computed tomography (CT) scan as part of a whole-body trauma assessment after a bicycle accident. Incidentally, a mass was detected between the left adrenal gland and the posterior wall of the gastric fundus. Given the differential diagnosis of either an adrenal tumor or a gastric diverticulum, laparoscopic resection was planned. Intraoperatively, the mass was identified as a gastric diverticulum, and a partial gastrectomy was performed. Histopathological examination revealed no abnormalities in the mucosa or the muscularis mucosae. However, the muscularis propria was markedly thinned and partially absent. There was no evidence of necrosis or inflammation. These findings are consistent with the segmental absence of intestinal musculature (SAIM) of the stomach. To our knowledge, this is the first reported case of SAIM of the stomach associated with a gastric diverticulum in an adult.

## Introduction

Segmental absence of the intestinal musculature (SAIM) is characterized by a complete loss of the muscularis propria (the muscle layer of the wall), with or without thinning, in a segment of the gastrointestinal tract [1-10]. SAIM was first recognized as a pediatric condition, typically presenting with intestinal obstruction, perforation, or intussusception (telescoping of the intestine) [1-6]. Since 1997, cases in adults have also been reported, most commonly presenting with spontaneous perforation in the duodenum, small intestine, or colon [7-10]. The etiology of SAIM remains unclear. Both congenital causes, involving developing failure of the muscular layer during fetal development, and acquired postnatal causes have been proposed [1-5,7,9].

Although rare, several cases of esophageal SAIM, characterized by a complete loss of the muscular layer of the esophageal wall, have been identified in adults during endoscopic submucosal dissection (ESD), highlighting the importance of recognizing this condition during the procedure [11,12]. In contrast, only one case of gastric SAIM in an adult has been reported to date, which was also incidentally discovered during ESD [13]. While a few cases of neonatal gastric SAIM have been described, almost all were associated with spontaneous gastric rupture [14-17]. Here, we report another adult case of gastric SAIM incidentally identified in association with a gastric diverticulum (sac-like bulge). To our knowledge, this is the first reported case of gastric SAIM associated with a gastric diverticulum in an adult.

## Case presentation

**Figure 1:**
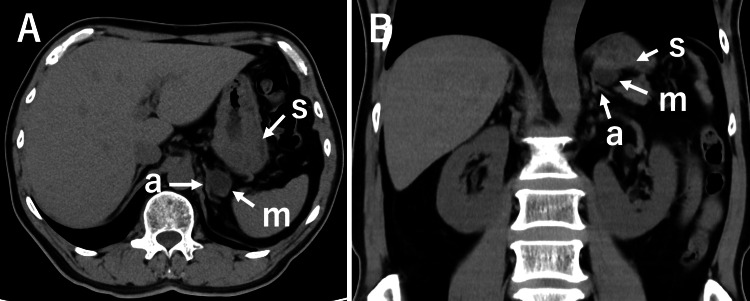
Axial (A) and coronal (B) cross-sectional CT images. The letters s, a, and m denote the stomach, left adrenal gland, and a mass shadow, respectively. A mass (m) is observed adjacent to the stomach (s) and the left adrenal gland (a).

A male patient in his late 50s presented to the emergency department after a forward fall from his bicycle due to sudden braking. He sustained significant blunt trauma to various parts of his body. His medical history included hypertension, with no other notable comorbidities or prior surgeries. He complained of no upper gastrointestinal symptoms. CT scans of the head, chest, and abdomen were performed to evaluate for traumatic injuries, and no fractures or acute internal injuries were detected. During the abdominal CT, a mass was incidentally discovered between the left adrenal gland and the posterior wall of the gastric fornix (Figure 1).

**Figure 2:**
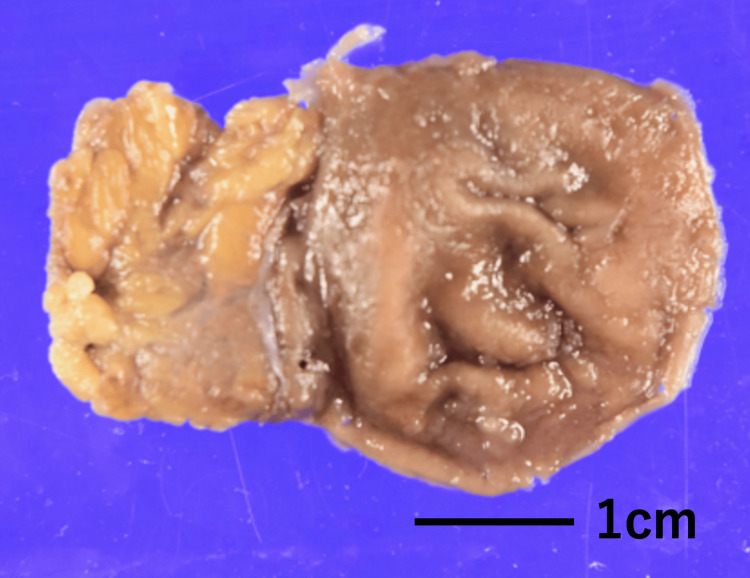
Macroscopic appearance of the gastric diverticulum resected during surgery.

The differential diagnosis included an adrenal tumor or a gastric diverticulum. Laparoscopic exploration was performed 2 months later with the intention of removing the mass. Intraoperatively, the mass was identified as a diverticulum arising from the posterior aspect of the stomach. Based on these findings, a diagnosis of gastric diverticulum was made, and a laparoscopic partial gastrectomy was successfully carried out. The diverticulum appeared as a sac-like bulge, with an inlet diameter of approximately 1.2 × 1.2 cm (Figure 2).

**Figure 3:**
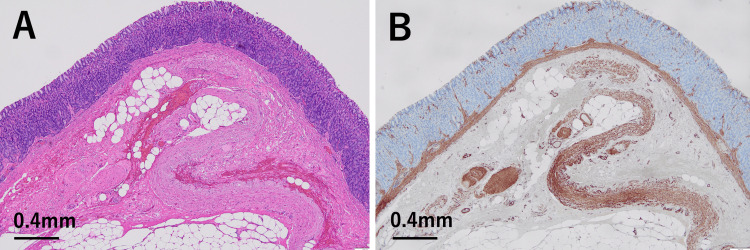
Histological and immunohistochemical findings in one area of the gastric diverticulum wall. (A) Hematoxylin and eosin (H&E) staining shows no abnormalities in the mucosa or the muscularis mucosae, with no evidence of necrosis and inflammation. Partial fibrosis and hemorrhage are observed in the submucosal tissue. (H&E stain, x4) (B) Immunohistochemistry for α-smooth muscle actin (α-SMA) demonstrates thinning of the muscularis propria when compared with the thickness of the muscularis mucosae, with partial loss of the muscularis propria. (α-SMA, ×4)

**Figure 4:**
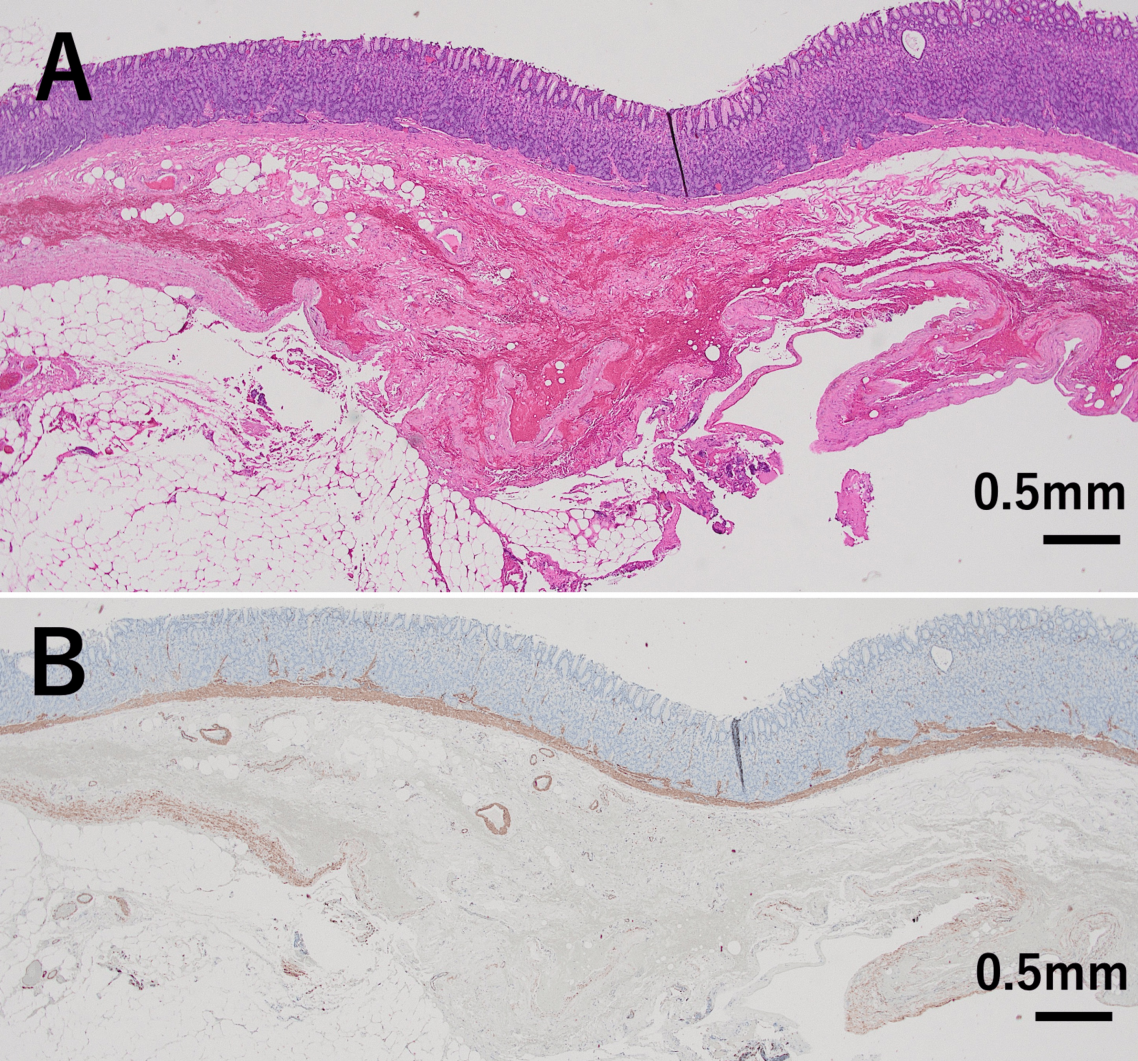
Histological and immunohistochemical findings in another area of the same gastric diverticulum wall. (A) Hematoxylin and eosin (H&E) staining shows no abnormalities in the mucosa or the muscularis mucosae, with no evidence of necrosis and inflammation. Focal fibrosis and hemorrhage are noted in the submucosal tissue. (H&E stain, x2) (B) Immunohistochemistry for α-smooth muscle actin (α-SMA) demonstrates marked thinning of the muscularis propria when compared with the thickness of the muscularis mucosae, with partial loss of the muscularis propria. (α-SMA, ×2)

The thickness of the bottom wall of the diverticulum was 2.5-4.0 mm. There was no evidence of adhesion between the diverticular wall and the surrounding tissue. No abnormalities were observed in the mucosa or the muscularis mucosae of the diverticular wall (Figures 3, 4).

A thin and sparse muscularis propria was preserved; however, in some areas, it was completely absent (Figures 3, 4). There was no evidence of necrosis or inflammation in the diverticular wall. Partial fibrosis and hemorrhage were observed in the submucosal area where the muscularis propria was absent, but not in other regions. Immunohistochemical staining for S-100 protein was performed, but no nerve plexuses were identified in the thin residual muscularis propria or in areas where it was absent.

## Discussion

The definitive diagnosis of SAIM relies on histological examination [2,5,6,9,10]. Characteristically, histological sections reveal a complete absence of the muscularis propria, occasionally accompanied by a thinned residual layer. The mucosa and muscularis mucosae remain anatomically intact, and no significant inflammation or necrosis is observed in the affected gastrointestinal wall. In the present case, histological examination of the gastric diverticulum revealed these characteristic features. Although fibrosis and hemorrhage were noted in the area lacking the muscularis propria, similar fibrotic changes have been previously reported in regions of muscular deficiency [4,18]. Therefore, the findings in this case are consistent with gastric SAIM associated with a gastric diverticulum. In this case, no nerve plexus was identified in areas where the muscularis propria was absent or thinned. The presence of a nerve plexus in such areas has been reported to vary among cases [6,9].

To our knowledge, only one case of gastric SAIM in an adult has been reported to date, which was found incidentally during ESD [13]. In that case, SAIM was suspected based on the synchronous movement of the submucosal layer with the patient’s breathing and was diagnosed using endoscopic ultrasound (EUS); however, histological confirmation was not obtained. In contrast, the diagnosis in our case was made histologically, based on examination of the gastric diverticulum. Therefore, this may represent the first histologically confirmed case of gastric SAIM associated with a gastric diverticulum in an adult.

Gastric diverticula are rare, with a reported prevalence of less than 2.6% [19]. They are classified into two types: true diverticula, which contain all layers of the gastric wall, and false diverticula, which do not. True diverticula account for approximately 70-75% of all gastric diverticula, are considered congenital in origin, and are typically located on the dorsal wall of the fundus, as in the present case [19]. In our case, the wall of the diverticulum showed a thinned or sparse muscularis propria with areas of complete loss. Therefore, if histological evaluation is limited to a small number of tissue sections, SAIM may easily be overlooked. Careful and extensive histological evaluation of multiple sections from the diverticular wall is likely essential for the accurate diagnosis of SAIM-associated diverticula. 

The etiology of SAIM remains unclear. Both congenital causes, involving developing failure of the muscular layer during fetal development, and acquired postnatal causes have been proposed [1-5,7,9]. Ischemia has been considered a potential contributing factor in both congenital and acquired causes [1-5,7,9]. It is supposed that ischemic injury may result in localized necrosis of the mucosa and muscularis propria, and that, due to the superior regenerative capacity of the mucosa, such damage could lead to segmental absence of the muscular layer [3-5]. Based on these considerations, two possible mechanisms may explain the formation of the gastric diverticulum associated with SAIM in this case. One is that congenital absence or thinning of the muscularis propria weakens the gastric wall, leading to outpouching. The other is that an acquired loss of muscularis propria occurred in a pre-existing congenital diverticulum located in the fundus, which is the most common site of gastric diverticula.

SAIM of the small and large intestines in adults has been considered a rare entity [7-9]. However, Tsuyuki et al. [10]recently reported that among 109 cases of intestinal perforation, excluding those caused by cancer invasion or appendicitis, 26 cases (24%) were attributable to SAIM, suggesting that the prevalence of this condition may be higher than previously assumed. Therefore, careful and extensive histological examination of spontaneous gastric perforation may reveal additional cases of gastric SAIM, although to date, only two cases, including the present case, have been reported.

## Conclusions

We present a rare case of segmental absence of intestinal musculature of the stomach associated with a gastric diverticulum incidentally detected in an adult. To our knowledge, this is the first histologically confirmed case of such an association in an adult. Awareness of this rare entity is important for accurate diagnosis, particularly in resected gastric diverticula.
